# Improvement of insulin sensitivity in diabetic and non diabetic patients with chronic hepatitis C treated with direct antiviral agents

**DOI:** 10.1371/journal.pone.0209216

**Published:** 2018-12-20

**Authors:** Alessandro Gualerzi, Mattia Bellan, Carlo Smirne, Margherita Tran Minh, Cristina Rigamonti, Michela Emma Burlone, Ramona Bonometti, Sara Bianco, Azzurra Re, Serena Favretto, Giorgio Bellomo, Rosalba Minisini, Gian Piero Carnevale Schianca, Mario Pirisi

**Affiliations:** 1 Department of Translational Medicine, Università del Piemonte Orientale UPO, Novara, Italy; 2 Division of Internal Medicine, “AOU Maggiore della Carità”, Novara, Italy; 3 Division of Internal Medicine, “Sant’Andrea Hospital”, Vercelli, Italy; 4 IRCAD, Interdisciplinary Research Center of Autoimmune Diseases, Novara, Italy; 5 Department of Health Sciences, Università del Piemonte Orientale UPO, Novara, Italy; University of Catanzaro, ITALY

## Abstract

**Background:**

The increased incidence of type 2 diabetes mellitus among hepatitis C virus (HCV) infected patients is likely due to viral-induced insulin resistance (IR). Indeed, control of diabetes in these patients benefits of successful antiviral treatment; whether the same applies to subtler alterations of glucose metabolism is unknown. We aimed to fill this gap.

**Methods:**

**The study population included** 82 HCV-RNA positive patients (48 males, median age 66 years, 73 with advanced fibrosis, 41 HCV-1b), attending the liver clinic of an academic hospital to receive direct antivirals. None was previously known to be diabetic. All underwent a standard oral glucose tolerance test (OGTT) before antiviral treatment and right after its conclusion.

**Results:**

At baseline, the majority of patients had evidence of abnormal glucose metabolism (N. = 45, 55%; impaired fasting glucose 10%, impaired glucose tolerance16%, both the above 12%, 17% diabetes), while only 37 (45%) were normally glucose tolerant (NGT). At the end of treatment, HCV-RNA quantification was below the detection threshold (HCV-RNA <12 UI/ml), for all patients enrolled. A significant decrease in glucose and insulin plasma concentrations was observed, leading to a significant reduction in Homeostasis Model Assessment (HOMA)-IR (from 3.42 [2.66–5.38] to 2.80 [1.78–3.95];p<0.001) and a corresponding increase in insulin sensitivity (ISI Belfiore from 0.49 [0.26–0.75] to 0.64 [0.42–0.91];p<0.001), despite a significant reduction in insulin secretion (EFP Stumvoll from 1363 [959–1730] to 1264 [976–1588];p = 0.027). Importantly, HOMA-IR reduction occurred also in the subgroup of NGT patients (p = 0.017). The number of NGT patients increased to 53, 65% (p = 0.013) paralleled by a reduced number of those satisfying criteria for prediabetic conditions (31 (38%) vs. 17 (21%); p = 0.025).

**Conclusions:**

Glucose metabolism parameters of HCV infected patients improve early after antiviral treatment, with benefits that are not limited to diabetics. These findings confirm how deep and widespread is the impairment of insulin pathways exerted by HCV infection.

## Introduction

The association between chronic hepatitis C virus (HCV) infection and insulin resistance (IR) is a major determinant of the increased incidence of type 2 diabetes mellitus (T2DM) in infected subjects [1]. Indeed, a meta-analysis involving a large number of patients attested that HCV infected patients incur in an additional risk of developing T2DM, whether compared to non-infected controls (OR 1.68) or to HBV-infected individuals (OR 1.80), suggesting a potential direct role of HCV in promoting diabetes [2]. HCV patients with pre-existing risk factors for T2DM have a 11-fold risk to develop the disease in comparison to non-infected controls with similar metabolic conditions [3]; furthermore, the viral induced IR affects the clinical course of liver disease, being independently associated to liver fibrosis progression [4]. When interferon-based regimens were used, abnormalities in glucose homeostasis affected treatment efficacy, with demonstrated lower percentages of viral eradication (sustained viral response, SVR) [5].

Viral-induced IR stems from both direct and indirect viral effects exerted on the insulin signaling pathway [6]. With regard to the former, the viral core directly promotes the proteasomal degradation of IRS-1 and IRS-2 through the induction of SOCS3, which leads to proteins ubiquitination, ending in reduced GLUT4 transmembrane expression [7]. As for the latter, the increased oxidative stress resulting from chronic infection enhances the expression of kinases involved in the development of IR, such as c-Jun N-terminal kinase (JNK) [8] and protein-phosphatase 2A (PP2A) resulting in insulin activity inhibition [9]. The final extra-hepatic IR propagation is caused by systemic spread of inflammatory factors, such as TNFα, IL-8, MCP-1, IL-18, produced in the liver in response to infection [10].

Several studies have attempted to determine if the metabolic disorders induced by HCV infection may regress after treatment, with conflicting results. An association between SVR after interferon-based treatment and Homeostasis Model Assessment index (HOMA-IR) reduction has been reported [11], and a recent retrospective study confirmed a significantly lower cumulative T2DM incidence in patients who obtained SVR [12]. Moreover, HCV patients with concomitant T2DM who obtain SVR show a reduced risk of developing diabetes complications compared to untreated HCV diabetics (end-stage nephropathy, acute coronary syndrome and stroke) [13]. However, these data have not been confirmed by others [14].

Since the introduction of anti-HCV regimens based on the new and highly efficacious direct antiviral agents (DAA) further observations have been made in the field. Retrospective studies have demonstrated early decreases in fasting blood glucose values and glycated hemoglobin (HbA1c), already detectable during the treatment course [15] and persisting long term [16]. Furthermore, after HCV clearance, many diabetic patients experience improved disease control leading—in some cases—to the need of reducing the doses of hypoglycemic drugs they use [17].

Currently available studies are either retrospective or did not investigate the glucose metabolism dynamically, thus precluding a detailed analysis of the changes related to viral eradication in patients not previously known as diabetics: this prospective study has been designed to fill these gaps.

## Methods

For this prospective cohort study, we enrolled consecutive patients with HCV-related chronic liver disease who attended the Liver Clinic of an academic hospital and were eligible for DAA treatment according to national rules. The patients underwent DAA treatment according to clinical indication as per routine care. Patients previously diagnosed as having T2DM were excluded. DAA regimens were chosen in accordance to the European Association for Liver Diseases (EASL) guidelines [18] (See S1 Table for more details). The study was conducted in accordance with the principles of the Declaration of Helsinki and approved by the Local Ethical Committee (Comitato Etico Interaziendale, “AOU Maggiore della Carità”, Novara). A written consent was obtained from all the participants.

All patients underwent a transient elastography (TE), assessing liver stiffness (LS), by FibroScan (Echosens, Paris, France), as previously reported [19]; N. = 66 (80%) had a repeat TE examination after the end of treatment. Detailed data on history and physical examination were recorded, along with the following laboratory tests, performed before initiating antiviral treatment and at the end of treatment (EoT, i.e. the day after the last day of treatment; 12 weeks or 24 weeks according to the different regimen chosen):

- Oral Glucose Tolerance Test (OGTT): this entails the ingestion of 75 gr of glucose and measurement of FPG, 1-h (1hPG) and 2-h plasma glucose (2hPG), measured along with the corresponding fasting, 1-h and 2-h plasma insulin concentrations (FPI, 1hPI and 2hPI). Glucose plasma concentration was measured by hexokinase (ADVIA, Siemens Healthcare, Germany; detection limit 4 mg/dl), while insulin was measured by chemiluminescence (Centaur, Siemens; detection limit 0.5μIU/ml). Patients were classified as having normal glucose tolerance (NGT), Impaired Fasting Glucose (IFG), Impaired Glucose Tolerance or T2DM, in agreement with the American Diabetes Association (ADA) criteria [20]. Subjects with IFG, IGT, IFG/IGT were lumped into a single group called prediabetes (preDM).
- HbA1c, assayed by high-pressure chromatography (“Variant Biorad II", Hercules, CA, USA), was interpreted according to ADA criteria (normal subjects: HbA1c<5.7%; preDM: ≥ 5.7% HbA1c < 6.5%; T2DM: HbA1c ≥ 6.5%). Since ribavirin affects erythrocyte half-life, changes in HbA1c values were evaluated only on the 62 patients whose treatment regimen did not include this drug.
- Circulating HCV Ribonucleic Acid (HCV-RNA) was researched with the diagnostic system of Amplicor HCV Test v2.0 (Roche Molecular Systems, Inc., Pleasanton, CA, USA), with sensitivity cut-off < 12 IU/ml.

Those patients who received a novel diagnosis of T2DM at baseline did not undergo a specific antidiabetic treatment, being only advised about hypoglycemic dietetic regimen. The OGTT-derived indices calculated both at baseline and at EoT were: Homeostasis model assessment (HOMA)-IR [21], Insulin Sensitivity Index according to Belfiore (ISI Belfiore) [22], Early First Phase according to Stumvoll (EFP Stumvoll) [22].

**Statistical analysis** was performed using Stata Rel. 15.1 (StataCorp LLC, College Station TX, USA). Data distribution was analyzed with the Shapiro-Wilk test. Continuous variables were analyzed by the Wilcoxon Test for paired data and the Mann-Whitney Test for comparison of independent groups; the existence of a correlation between them was verified calculating the Spearman correlation coefficient. The association between categorical variables was tested by the Pearson chi-square Test or the Fisher Exact Test, as appropriate. Logistic regression analysis was conducted to identify the independent predictor(s) among a set of variables displaying at univariate analysis a significant association with decrease of HOMA-IR following DAA treatment, defined as a reduction exceeding 20% of the baseline value. The threshold for statistical significance was 0.05 (two tails) for all tests used.

## Results

The study population included 82 subjects (48 males (59%), median age 66 [53–74] years). The median body mass index was 24.8 [22.2–27.7] kg/m^2^. Forty-two out of 82 (52%) were cirrhotics, while other 31 (38%) had advanced liver fibrosis. Furthermore, before starting DAA treatment, N = 45 patients with abnormal glucose metabolism of any kind (prediabetes or diabetes) accounted for 55% of the total study population.

**Fig 1:**
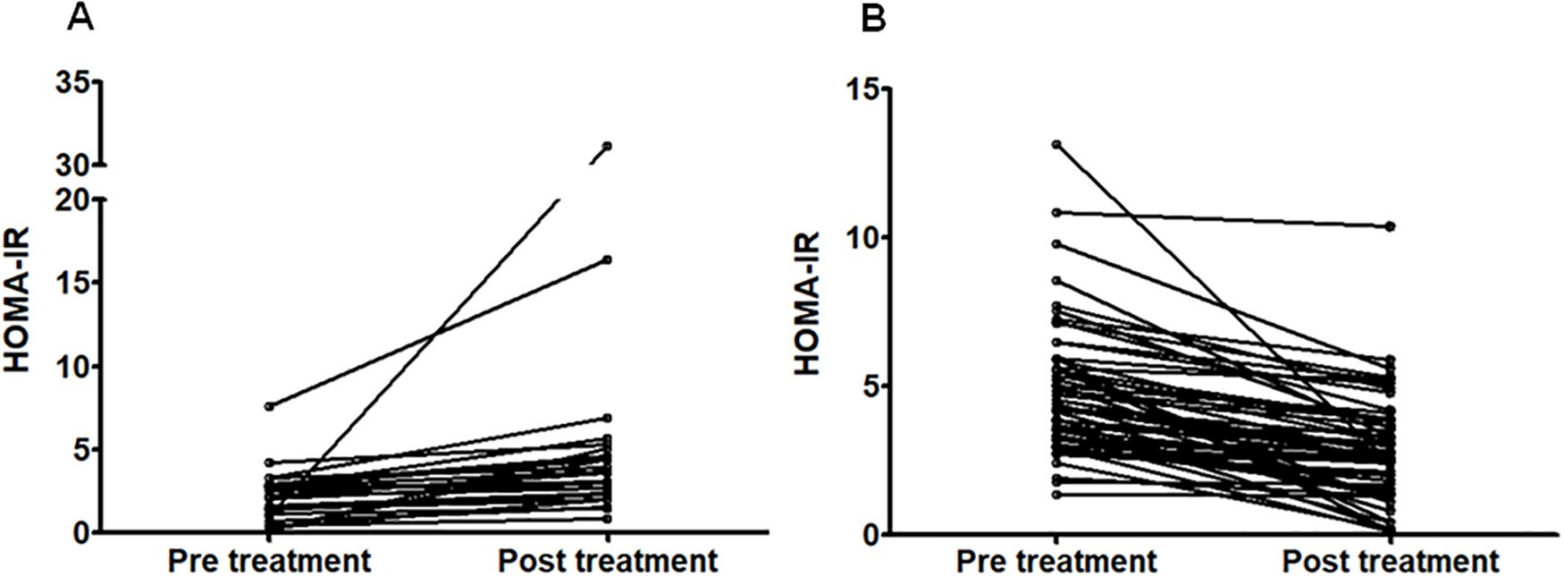
Individual homeostasis model assessment: Insulin resistance (HOMA-IR) values before and after antiviral treatment of hepatitis C. Panel A: patients whose HOMA-IR values increased after treatment (N. = 26); panel B: patients whose HOMA-IR values decreased after treatment (N. = 56).

At EoT, HCV RNA quantification was below the detection threshold in all cases; 81/82 (99%) patients achieved SVR. In Table 1 we report the changes observed on OGTT results after the antiviral treatment. A significant decrease in glucose and insulin plasma concentration is evident both in fasting conditions as well as after 60 and 120 min. Similarly, a significant variation of HOMA-IR, ISI and EFP Stumvoll was observed (also see Fig 1). HbA1c decreased from 5.5 [5.3–5.8]% to 5.4 [5.2–5.6]% (p = 0.008). As expected, a significant decrease in AST, ALT and GGT plasma concentration also occurred.

**Table 1:** Effects of DAA treatment on glucose metabolism and liver biochemistry.

Variables	Pre treatment	Post treatment	p values
**FPG (mg/dl)**	94 (87–104)	92 (87–98)	0.028
**1h-PG (mg/dl)**	186 (137–219)	156 (121–208)	<0.001
**2h-PG (mg/dl)**	132 (106–179)	114 (91–142)	<0.001
**FPI (IU/ml)**	16 (11–22)	12 (8–17)	0.002
**1h-PI (IU/ml)**	108 (55–166)	82 (50–126)	<0.001
**2h-PI (IU/ml)**	98 (55–182)	74 (41–121)	<0.001
**HbA1c (%)**	5.5 (5.3–5.8)	5.4 (5.2–5.6)	0.008
**HOMA-IR**	3.42 (2.66–5.38)	2.80 (1.78–3.95)	<0.001
**ISI Belfiore**	0.49 (0.26–0.75)	0.64 (0.42–0.91)	<0.001
**EFP Stumvoll**	1363 (959–1730)	1264 (976–1588)	0.027
**AST (IU/L)**	55 (37–102)	26 (20–30)	<0.001
**ALT (IU/L)**	73 (40–136)	20 (15–27)	<0.001
**GGT (IU/L)**	55 (32–121)	25 (16–37)	<0.001

Abbreviations: FPG, Fasting Plasma Glucose; 1hPG, 1-h plasma glucose after the glucose challenge; 2hPG, 2-h plasma glucose after the glucose challenge; FPI, fasting plasma insulin; 1h-PI, 1-h plasma insulin concentration after the glucose challenge; 2hPI, 2-h plasma insulin concentration after the glucose challenge; HbA1c, glycated hemoglobin; HOMA-IR, Homeostasis Model Assessment index; ISI Belfiore, Insulin Sensitivity Index according to Belfiore; EFP Stumvoll, Early First Phase according to Stumvoll; AST, Aspartate aminotransferase; ALT, Alanine aminotransferase; GGT, Gamma-glutamyl transferase.

There was no significant change in body weight at baseline (70 [60–82] kg) vs. EoT (71 [59–80]) (p = 0.276); in fact, body weight decreased in 29/82 patients (35%), remained unchanged in 31/82 (38%) and increased in 22/82 (27%). Fifty-five patients showed a decrease in HOMA-IR, while in 27 subjects HOMA-IR increased from baseline (Fig 1). No trend to decreased HOMA-IR was observed in relationship to weight change before vs. after treatment (p = 0.982). Specifically, the differences observed in HOMA-IR after vs. before treatment were -0.95 [-2.24–0.49] among those in whom body weight increased, -0.60 [-1.91–0.49] among those in whom body weight remained unchanged, and -1.06 [-1.97–0.92] among those whose body weight decreased. Considering patients to have decreased their HOMA-IR if it decreased >20% after treatment compared to baseline, N. = 40 (49%) passed the threshold, while N.42 (51%) either remained stable or worsened their HOMA-IR. Table 2 displays a comparison of these two subgroups. As shown, those patients who experienced a decrease of HOMA-IR after treatment had at baseline a significantly lower BMI, higher HOMA-IR, higher plasma insulin both at fasting and after glucose challenge, lower insulin sensitivity and higher Stumvoll early first phase. Notably, liver stiffness after treatment (measured in N. = 66 patients, 80%) decreased >30% in comparison to baseline in 36/66 (54%), among whom N. = 18 (50%) belonged to the group in which HOMA-IR decreased significantly (p = 0.810). At logistic regression, conducted having significant improvement of HOMA-IR after treatment as dependent variable, and baseline BMI, HOMA-IR, fasting plasma insulin, plasma insulin 2h after glucose challenge, ISI Belfiore and Stumvoll early first phase as predictive variables (N. of observations = 82; logistic regression chi-square = 41.2), the baseline BMI was the only independent predictor identified (OR 0.66, 95% confidence interval 0.52–0.83, p <0.001).

**Table 2:** Comparison of baseline characteristics of patients based on HOMA-IR modifications after treatment (those in whom a decrease >20% was observed vs. all others).

Variables	HOMA-IR after treatment		p values
	Decreased (N. = 40)	Stable or increased (N. = 42)	
**Age (years)**	67 (56–75)	65 (52–74)	0.391
**BMI (kg/m**^**2**^**)**	23.8 (22.1–26.1)	25.8 (23.6–29.1)	0.040
**FPG (mg/dl)**	95 (89–110)	93 (86–101)	0.106
**2h-PG (mg/dl)**	141 (106–183)	131 (103–179)	0.687
**FPI (IU/ml)**	19 (15–24)	12 (7–16)	<0.001
**2h-PI (IU/ml)**	135 (61–100)	82 (50–136)	0.025
**ISI Belfiore**	0.38 (0.22–0.63)	0.57 (0.35–0.87)	0.019
**EFP Stumvoll**	1528 (1234–1943)	1142 (880–1585)	0.012
**HbA1c (%)**	5.4 (5.2–5.7)	5.5 (5.3–5.7)	0.462
**HOMA-IR**	4.54 (3.42–5.91)	2.73 (1.51–3.57)	< 0.001
LS (kPa)*			
**Before treatment**	11.6 (10.2–17.3)	12.5 (10.4–15.7)	0.981
**After treatment**	7.6 (5.9–12.8)	8.0 (6.8–11.4)	0.812
**AST (IU/L)**	63 (43–99)	49 (35–105)	0.539
**ALT (IU/L)**	74 (41–111)	64 (40–147)	0.897
**GGT (IU/L)**	56 (37–121)	55 (31–121)	0.792

Abbreviations: BMI, Body Mass Index; FPG, Fasting Plasma Glucose; 2hPG, 2-h plasma glucose after the glucose challenge; FPI, fasting plasma insulin; 2hPI, 2-h plasma insulin concentration after the glucose challenge; HbA1c, glycated hemoglobin; HOMA-IR, Homeostasis Model Assessment index; ISI Belfiore, Insulin Sensitivity Index according to Belfiore; EFP Stumvoll, Early First Phase according to Stumvoll; AST, Aspartate aminotransferase; ALT, Alanine aminotransferase; GGT, Gamma-glutamyl transferase.

* Data missing in N. = 16 patients.

Even considering separately the subgroup of patients in whom HOMA-IR remained stable or increased, both plasma glucose 2h after the glucose challenge and HbA1c improved significantly to median values of 114 mg/dl [91–133] (p = 0.021) and 5.4% [5.2–5.7] (p = 0.036), respectively.

Table 3 shows patients’ stratification accordingly to their glucose metabolism status, before and after antiviral treatment. It demonstrates that, after DAA, the percentage of NGT patients increased substantially, in parallel to the decrease of pre-diabetics. Moreover, two patients, formerly categorized as diabetics, did not satisfy OGTT criteria for T2DM anymore.

**Table 3:** Changes in the glucose metabolism status of patients.

Class of glucose tolerance	Pre Treatment N (%)	Post Treatment N (%)
**NGT**	37 (45)	53 (65)
**PreDM**	31 (38)	17 (21)
**T2DM**	14 (17)	12 (15)

Data are shown as frequencies (%); p values refer to Pearson chi-square test. Abbreviations: NGT, Normal glucose tolerance; IFG, Impaired fasting glucose; IGT, Impaired glucose tolerance; PreDM, IFG and/or IGT.

Changes of HOMA-IR in the entire population were not correlated to changes in the plasma concentrations of ALT (Spearman Rho coefficient = -0.018; p = 0.873), AST (Spearman Rho coefficient = -0.117; p = 0.299), GGT (Spearman Rho coefficient = -0.221; p = 0.058), or changes in liver stiffness after treatment (Spearman Rho coefficient = -0.199, p = 0.109). Moreover, the viral load at baseline did not show any correlation with changes of HOMA-IR after treatment (Spearman Rho coefficient = -0.154; p = 0.166). In turn, no correlation existed between changes in HOMA-IR and either age (Spearman Rho coefficient = -0.058; p = 0.604) or weight change before vs. after treatment (Spearman Rho coefficient = -0.011; p = 0.925). N- = 53 out of 82 (64%) patients underwent a sofosbuvir based regimen, vs. 29/82 (36%) for whom a protease inhibitor based regimen was used: there was no significant difference in the change of HOMA-IR observed in these two groups (p = 0.812). Finally, HOMA-IR was shown to decrease significantly after vs. before treatment even when the 37 patients with baseline normal glucose tolerance status were analyzed separately (p = 0.017).

## Discussion

To study alterations of glucose metabolism, FPG—on which previous studies dealing with changes induced by DAA treatment were based [15]–is a rather insensitive index. Here, instead, we took advantage of the OGTT to have high sensitivity, accurate categorization of patients, and clear demonstration of the precocity of changes induced by DAA treatments.

Interestingly, more than half of our study population showed an abnormal glucose metabolism at baseline; 17% of them, not previously known to be diabetics, satisfied criteria for T2DM. These rates of abnormal glucose metabolism are significantly higher than in previous studies [23], likely due to the higher sensitivity of an OGTT-based approach. HCV infection appears to have a major impact on the response to glucose load, possibly because the virus might significantly affect muscular IR (which characterizes IGT) besides hepatic IR (which characterizes IFG) [24]. In support to this hypothesis, 11/14 received a novel diagnosis of T2DM because of the 2hPG value, while FPG and HbA1C were far less sensitive (21.4% and 14.3% respectively). This observation suggests that the rate of missed T2DM diagnosis might be higher than expected in HCV-infected subjects, in the absence of targeted assessments with OGTT.

At EoT, we observed a significant reduction in IR and a corresponding increase in insulin sensitivity, in line with a recent paper reporting significant improvements of HOMA-IR among 102 diabetic patients who achieved SVR [25]. Interestingly, these improvements extend to NGT subjects, suggesting that the benefits of viral eradication cover the entire spectrum of glucose metabolism, not just diabetes. Finally, the improvement in glucose homeostasis is so relevant to lead to a significant decrease of HbA1c, despite the brevity of treatment. The explanation of these effects is increased sensitivity to insulin, since insulin secretion (assessed by EFP) is reduced. Careful monitoring of patients undergoing hypoglycemic drugs or insulin therapy and/or early specialist referral is thus advised to avoid hypoglycemia. Our findings replicate similar results recently obtained by another Italian group, that reported an improvement in insulin sensitivity and a reduction of pancreatic secretion at the end of DAA treatment and after 3 months of follow-up in a group of HCV positive, non-diabetic patients with advanced liver fibrosis [26]. Interestingly, we also report that the patients who mostly benefit from DAA treatment in terms of glucose metabolism are those with a higher baseline HOMA-IR; however, even in the subset of patients where HOMA-IR did not improve significantly, DAA treatment led to a significant reduction of HbA1c and post-OGTT plasma glucose concentration. We do not deny that many other factors must be taken into account besides viral infection when interpreting parameters of glucose homeostasis in these patients. One could speculate, for example, that an improvement in perceived well-being during treatment may have favoured the adoption by these patients of a more active lifestyle. Even in this notoriously unlikely event, however, the changes observed in the glucose metabolism of the patients awe studied re not explained by weight loss during treatment. On the other hand, the present study suggests that a significant HOMA-IR reduction can confidently be predicted to occur among patients who undergo DAA starting with high baseline HOMA-IR, despite not being overweight or obese. Indeed, out of 19 patients of the present series who had BMI <25 kg/m^2^ and HOMA-IR >3.4, only two did not pass the threshold of 20% reduction following treatment. This is hardly surprising, since the abovementioned specifics identify patients for whom the virus plays a major role in determining IR.

One of the main limitations of our study belongs to its small sample size, with a study population mostly composed of patients with advanced liver fibrosis, which makes our findings not automatically extendible to all HCV-infected patients. Moreover, another potential confounder is related to the heterogeneity of viral genotypes distribution in our population. Genotype has been proposed as a major determinant for the association of HCV infection and glucose metabolism alteration, although this aspect is still a matter of debate [27]. However, we believe that data are solid enough to alert the clinicians on the possibility of hidden T2DM among HCV-infected individuals despite normal FPG, as well as of the benefits provided by HCV clearance on glucose homeostasis at large. It has to be stressed that our study was designed to verify the existence of early modifications of glucose metabolism following DAA treatment. In our opinion, this is a strength, not a weakness of the study, because—by showing how quickly insulin sensitivity is restored after treatment—the results confirm how deep and widespread is HCV interference on insulin pathways. However, we are not allowed any inference on putative long term benefits provided by viral eradication on glucose metabolism. Indeed, further studies will be required to clarify whether eliminating HCV infection by means of DAA treatment may result in either preventing or delaying the development of T2DM in HCV positive subjects.

In conclusion, HCV patients treated with DAA undergo a marked reduction of IR. The precocity of this phenomenon and its widespread expression among diabetics as well as non-diabetics support the hypothesis of a paramount role of the virus in blocking the insulin signaling pathways.

## Supporting information

S1 Table Therapeutic regimen administered. The total number of patients and the relative percentage are shown. For abbreviation: RBV, Ribavirin.
(DOCX)

S1 Database We have included the anonymized version of our database as supporting material. (XLSX)
