# Supplementary material for: Improvement of insulin sensitivity in diabetic and non diabetic patients with chronic hepatitis C treated with direct antiviral agents
Source: PLoS One. 2018 Dec 20;13(12):e0209216. doi: 10.1371/journal.pone.0209216 (PMC6301649; doi:10.1371/journal.pone.0209216)
Supplement: S1 Table — The total number of patients and the relative percentage are shown. For abbreviation: RBV, Ribavirin. (DOCX) [file pone.0209216.s001.docx]

| Therapeutic Regimens | Total | With RBV | Without RBV |
| --- | --- | --- | --- |
|  | n. (%) | n. (%) | n. (%) |
| Sofosbuvir | 6 (7.3) | 6 (7.3) | 0 (0.0) |
| Sofosbuvir/ledipasvir | 25 (30.5) | 10 (12.2) | 15 (18.3) |
| Sofosbuvir + daclatasvir | 17 (20.7) | 4 (4.9) | 13 (15.8) |
| Paritaprevir/ritonavir/ombitasvir + dasabuvir | 18 (22.0) | 0 (0.0) | 18 (22.0) |
| Elbasvir/Grazoprevir | 11 (13.4) | 0 (0.0) | 11 (13.4) |
| Sofosbuvir/Velpatasvir | 5 (6.1) | 0 (0.0) | 5 (6.1) |
